# National Health Insurance Notes

**Published:** 1924-08

**Authors:** 


### NATIONAL HEALTH INSURANCE NOTES.

### APPOINTMENT OF ROYAL COMMISSION.

The *personnel* of the Royal Commission on National Health Insurance is announced. The terms of reference are: "To inquire into the scheme of National Health Insurance established by the National Health Insurance Acts, 1911-1922, and to report what, if any, alterations, extensions or developments should be made in regard to the scope of that scheme and the administrative, financial and medical arrangements set up under it." The Commission will be constituted as follows: Lord Lawrence of Kingsgate (Chairman), Sir John Anderson, Sir Humphrey Rolleston, Bt., Sir Alfred Watson, Sir Arthur Worley, Sir Andrew Duncan, Mr. A. D. Besant, Mr. Fred. Bramley, Professor Alexander Gray, Miss Gertrude Tuckwell, Mrs. Harrison Bell, Mr. James Cook, Mr. John Evans, and Mr. William Jones. The Secretary to the Commission, to whom all communications should be addressed, is Mr. E. Hackforth, of the Ministry of Health, Whitehall. S.W. 1; and the Assistant Secretary is Mr. J. W. Peck, of the Scottish Board of Health.

### The Lack of Medical Representation.

In the composition of the Commission there is a noteworthy absence of any official element from the Ministry of Health. The inclusion of that distinguished Civil Servant, Sir John Anderson, at one time Secretary of the National Health Insurance Commission, and of Professor Alexander Gray, a former high official of that body, is a guarantee of strength and sound direction in assisting the Chairman on the lines along which the Commission will proceed; we glanced at some of their problems in our June issue. Sir Alfred Watson, the Government Actuary, is in a class by himself, and no Commission could proceed far without his assistance, while so far as the Secretary, Mr. Hackforth, is concerned, if there is anything which he lacks in his knowledge of the working of the National Health Insurance scheme, a closer acquaintance with it is clearly not worth while. It would be idle to pretend that the composition of the Commission has given satisfaction in what must be considered interested quarters. The British Medical Association, through their *Journal*, are outspoken in their criticism of the almost complete absence of medical representation, while recognising the value of the inclusion of the distinguished President of the Royal College of Physicians. The societies are also dissatisfied. It is a matter for regret, as we have on occasion observed, that doctors and societies must be regarded as opposite camps. That, however, is the situation, and the Minister of Health will doubtless find solace in the reflection that, as both doctors and societies are dissatisfied, he has fairly succeeded in setting up an entirely impartial and independent body. It has big work in front of it with far-reaching results on the future of the national health.

### Range of Medical Treatment.

A question of interest and importance has been decided by a Court of Referees under the Medical Benefit Regulations of the National Insurance Acts. The inquiry dealt with the question whether or not treatment by radiant heat is within the range of medical service to be rendered by panel practitioners under their terms of service. In the case before the Court, an insured person was treated by radiant heat for chronic rheumatism of the left shoulder. The treatment involved numerous applications, of about thirty minutes each, of radiant heat from a 100-c.p. carbon lamp on a circuit of 240 volts, the practitioner having the necessary apparatus. The Insurance Committee had contended that the treatment does not require skill more than should be expected from an ordinary practitioner, that the necessary skill can soon be acquired and is not comparable with that needed for many operations commonly undertaken by panel practitioners, and that the fact that the treatment requires special apparatus is not relevant, as the same argument would apply to other services which are admittedly within the range of the medical service.

### The Referee's Decision.

The Court concluded, not without some doubt, that this service is outside the range of ordinary medical service, and its decision was influenced by four main factors—that the treatment is not taught or regarded as part of the ordinary medical curriculum; that while the actual manipulation of the apparatus is without difficulty, the experience requisite for the efficient application of the treatment would not ordinarily be obtained by the insurance practitioner; that the time involved in giving the treatment is such that not many private practitioners would find it worth while to instal the apparatus for the treatment of private patients; and that the contrary conclusion would involve the insurance practitioner in the expense of installing the apparatus, and the Court did not think that the Acts and Regulations intended to impose on panel doctors the burden of undertaking in their insurance practice a duty of this kind. The Court guarded itself against expressing the opinion that it would be impossible for general practitioners to attain the skill necessary, given the opportunity and willingness to undertake the necessary study and expense. The Court added that its decision involved no reflection on the Insurance Committee in raising the issue.

### A Limited Service.

The scope of the Medical Benefit service for insured persons is already very limited, and this case serves as a further illustration, if it is needed, of the restrictions from which this service suffers. The service, it would seem, is limited not merely by the range of skill of the general practitioners as a whole, but by the extent of the equipment of the individual practitioner. It is much to be hoped that, as the result of the enquiries and recommendations of the Royal Commission, this defect will be remedied.

